# Clinical effects of cognitive behavioral therapy in heart failure patients: a meta-analysis of randomized controlled trials

**DOI:** 10.1186/s12906-023-04117-2

**Published:** 2023-08-07

**Authors:** Mahmoud Balata, Mohamed Ibrahim Gbreel, Asmaa Ahmed Elrashedy, Ralf Westenfeld, Roman Pfister, Sebastian Zimmer, Georg Nickenig, Marc Ulrich Becher, Atsushi Sugiura

**Affiliations:** 1 Department of Internal Medicine and Polyclinic II, University Hospital Bonn, Venusberg-Campus 1, 53127 Bonn, Germany; 2 Faculty of Medicine, October 6 University, Giza, Egypt; 3 Faculty of Medicine, Kafr El-Shaikh University, Kafr El-Shaikh, Egypt; 4 Department of Internal Medicine and Cardiology, University Hospital Duesseldorf, Duesseldorf, Germany; 5 Department of Internal Medicine and Cardiology, University Hospital Cologne, Cologne, Germany

**Keywords:** Anxiety, Cognitive behavioral therapy, Depression, Heart failure care, And Meta-analysis

## Abstract

**Background:**

About 20–40% of people with Heart failure (HF) suffer from some depression, which is 4–5% greater than the overall population. This depression can lead to undesirable outcomes, including elevated mortality rate and frequent hospitalization.

**Purpose:**

The current study aims to evaluate the impact of cognitive behavioural therapy (CBT) on self-care and the symptoms of depression and anxiety in HF patients.

**Methods:**

We searched PubMed, Web of Science (WOS), Scopus, and Cochrane Library till 15 October 2022. All relevant randomized controlled trials (RCTs) were included. The data were extracted and pooled using Review Manager software (RevMan 5.4). Continuous data were pooled as mean difference and 95% confidence interval (CI).

**Results:**

Our search retrieved 1146 records, and 7 studies (611 patients) were finally included. We assessed the Beck Depression Inventory-II (BDI-II) as the primary outcome of the study. Hamilton Rating Scale for Depression (HRSD-17), Change in Beck Anxiety Inventory, Kansas City Cardiomyopathy Questionnaire (KCCQ), and Self-Care of Heart Failure Index (SCHFI) were also assessed as secondary outcomes. With CBT, BDI-II showed a significant reduction after 4 to 6 months follow-up (MD = -4.87, 95% CI: [-8.06; -1.69], P *= 0.003*) as well as 8 to 9 months follow-up (MD = -5.71, 95% CI: [-8.95; -2.46], *P = 0.0006*). But no significant difference was shown with 3 months follow-up (M.D=-4.34; 95%CI: [-10.70; 2.03], P = 0.18).

**Conclusions:**

CBT has long-term (4–9 months) significant favorable outcomes decreasing anxiety and depression compared to non-CBT groups. No significant short-term (less than 3 months) impact on HF patients’ self-care, depression, or anxiety were shown.

## Introduction

Heart failure (HF) and other cardiovascular diseases (CVDs) are major health problems in our modern world. Globally, it is reported that over 64 million people had HF, and over 18.5 million died from CVDs only in 2019 [1].

The etiology of such a complicated and progressive disease as HF is mainly due to any structural or functional cardiac defect resulting in failure or the inability of the ventricles to fill or expel blood [2]. This disease significantly places a big load on patients, health care providers, and the overall health care systems. It is related to undesirable outcomes such as elevated mortality rate, frequent hospitalization, and severe physical injury. In addition to physical problems, this disease is also related to psychological suffering, including mainly depression and anxiety [3].

Aside from the physiological level of HF, several reports [4–6] have indicated that the primary significant risk factor for disability and death due to HF is depression. In HF patients, a three-month depressive episode doubles the risk of death and triples the likelihood of re-hospitalization within a year [7]. Additionally, people with HF who are depressed have lesser capacities for self-care than those who are not depressed. Between 9% and 60% of people who have HF also have depression, which is a relatively high prevalence [4, 5]. This high prevalence is multifactorial and may be brought on by debilitating symptoms, physical function limitations, future insecurity, lack of confidence in the ability to play personal, social, and professional roles, concerns regarding engaging in some activities, and lack of self-esteem [6].

Besides depression, death anxiety is among the most critical psychological stresses for people with HF. This anxiety, in turn, aggravates the depressive state of the patient and worsens the patient’s overall quality of life [8]. Hence, mental health therapies and supportive care for hospitalized patients seem relevant and necessary given the high rate of depression and death anxiety among HF patients [9].

Despite adopting the usage of selective serotonin reuptake inhibitors (SSRIs) in HF patients with depression, the effectiveness of these medications in HF patients is still controversial [10]. Therefore, to treat patients with heart failure, it appears to be an even greater need to adopt psychological treatments. By selecting the most efficient therapy with the fewest adverse events, therapists can boost the probability of recovery in HF patients who are depressed [11]. As a result, specialized psychological therapies, such as cognitive-behavioural training (CBT), have emerged.

CBT is a proactive, planned, time-restricted, and goal-directed method that uses cognitive-behavioural techniques to address patients’ issues. This therapy is a progressive procedure that aids a patient in gradually changing their behaviour in order to lessen the mental burden that the illness has had on them [12]. Several trials have highlighted the importance of psychosocial aspects in patients with heart failure receiving cognitive-behavioural treatment [10, 13]. Yet, only a few trials utilized psychological therapy with brief cognitive-behavioural methods on depressed HF patients to lessen depression and anxiety and enhance self-care practices [4, 14]. In this context, the current pooled analysis aims to evaluate the impact of CBT on self-care and the symptoms of depression and anxiety in HF patients.

## Materials and methods

This systematic review and meta-analysis was conducted according to the guideline of the Cochrane Handbook for systematic reviews of interventions [15] and then was reported using the latest version of preferred reporting items for systematic review and meta-analysis (PRISMA-P statement) [16].

### Literature search

Four databases (PubMed, SCOPUS, Web of Science (WOS), and Cochrane Library) were searched till 15 October 2022 using the following search strategy: (“Cognitive Behavioral Therap*” OR “Cognitive Behaviour Therap*” OR “Cognitive Therapy” OR “Cognitive Behavior Therap*” OR “Cognitive Psychotherapy” OR “Cognitive Psychotherapies” OR “Cognition Therapy” OR “Cognition Therapies” OR) AND (“Heart Failure” OR “Cardiac Failure” OR “Myocardial failure” OR “Heart Decompensation” OR “Cardiac infraction” OR “Myocardial infraction”). All references of the included studies were screened for eligibility.

### Eligibility Criteria and Study Selection

The Population, Intervention, Comparator, Outcomes, and Study designs (PICOS) criteria for including studies were restricted to the following criteria: (P) Hart failure patients (I) CBT (C) Any comparator (O) Depression-related outcomes (S) Randomized controlled trials (RCTs). On the other hand, we excluded studies with different cardiovascular patients groups other than hear failure, digital CBT, non-English studies, studies with irrelevant outcomes, and different study designs other than RCTs including case reports, conference abstracts, case series, letters to the editor, author opinion papers, and review articles. Four independent authors screened the title and abstract to include relevant studies, then the full text to confirm the eligibility to be finally included. In cases of indecision, a senior author was consulted to confirm the decision.

### Primary and secondary endpoints

Beck Depression Inventory II (BDI-II) was assessed as the primary outcomes of the study. Hamilton Rating Scale for Depression (HRSD-17), Change in Beck Anxiety Inventory, Kansas City Cardiomyopathy Questionnaire (KCCQ), Self-Care of Heart Failure Index (SCHFI) were also assessed as secondary outcomes.

### Data extraction

We extracted the characteristics of included studies, baseline characteristics of included patients, risk of bias domains, and study outcomes. The extracted characteristics included of the included studies included the study first author, year of publication, protocol registration number, sample size, setting, inclusion criteria, number of CBT sessions, primary outcomes, and the follow-up duration. The patients’ characteristics were listed as follow: number of each group (CBT group and control group), gender, body mass index (BMI), marital status, employment, education, income (< 30,000 $/y), New York Heart Association (NYHA) classification (I, II, III, IV), odd of suffering from comorbidities including (previous MI, diabetes mellitus, prior stroke, chronic obstructive pulmonary disease [COPD], hypertension, renal diseases, history of atrial fibrillation [AF]), and Antidepressant medication use at admission.

### Quality assessment

The Cochrane Risk of Bias tool [17] was used to assess the quality of the included RCTs. The ROB tool includes six domains to assess the following domains: selection bias, performance bias, detection bias, attrition bias, reporting bias, and other potential biases. Two authors independently assessed the quality of included RCTs and categorized the studies into three categories: “low risk”, “high risk”, or “unclear”. Any disagreements were resolved through discussion and consensus with a senior author.

### Data synthesis

We conducted this meta-analysis by using two programs. We used RevMan software (Version 5.4 for Windows) (Nordic Cochrane Center, Copenhagen Denmark, 2014). As the whole outcomes in the study wer continuous outcomes, we presented all data as mean difference with 95% confidence intervals (CIs). We tested the heterogeneity between pooled studies using chi-square and I-square tests. When the heterogeneity between studies at a P-value (chi-square) of < 0.1 and I² > 50%, we used a random-effect model for analysis. We performed subgroup analysis to test whether the effect estimate of Vilazodone differs significantly according to the dose and duration. According to Eggar et al. [18], publication bias assessment via generating a funnel plot couldn’t be performed due to the limited number of the included studies (less than 10 trials).

## Results

### Search results

**Fig. 1:**
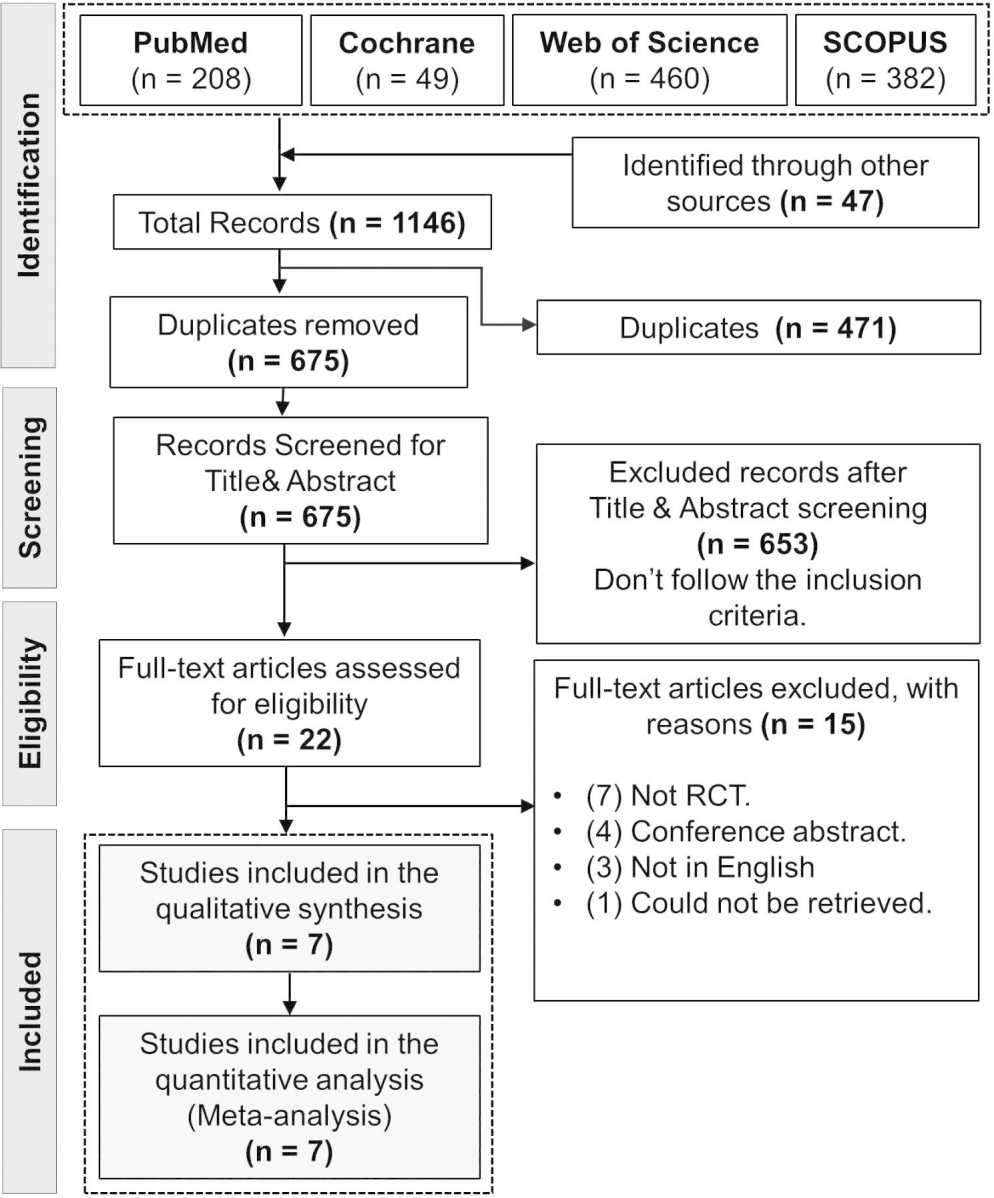
PRISMA flow diagram of the literature search results

We conducted a comprehensive search in four online databases including PubMed, the Cochrane Library, Web of Science, and SCOPUS. Our search retrieved 1146 records; 471 records were removed due to duplication. 22 studies were eligible after the title and abstract screening, and 7 studies were finally included [19–25]. Six studies [20–25] of them were included in the quantitative meta-analysis (Fig. 1).

### Characteristics of the included studies

Regarding the site of included trials, four trials were conducted in the USA [20–23]. Two studies were conducted in Iran [24, 25], and one study was conducted in the Philippines [19] The least follow-up period was one month [25], and the most extended follow-up period was 32 weeks [22]. Further details regarding CBT details and studies’ conclusion are shown in Table 1. The mean age of patients was more than 50 years in all studies, and the percentage of males was more than 50% in all studies. The percentage of married patients ranged between 37% and 90%. Regarding NYHA classification, degrees II and III were the most common. Further details are shown in Table 2.

**Table 1:** Summary of the included studies

Study ID	Study registration	Sample Size	Setting	Inclusion criteria	Number of CBT sessions	Control	Primary outcomes	Follow up
**Cajanding et al. 2016**	N/R	100	Philippines	(1) Adults hospitalized patients with HF (2) Not for other co-morbidities.	Once weekly for 12 weeks	Usual care	Quality of life, self-esteem, and mood scores	13 weeks
**Dekker et al. 2012**	NCT01275742	42	USA	(1) Patients with 21 years or older admitted with HF at 2 Lexington, Kentucky, hospitals (2) Patients with preserved or non-preserved systolic HF were eligible.	A single, 30-minute, one-on-one session	Usual care	Depressive symptoms	13 weeks
**Freedland et al. 2015**	NCT01028625	158	USA	(1) Patients diagnosed with HF 3 months or less before the screening (2) Current severe depressive episode, and a “depressed” Beck Depression Inventory score (BDI-II) (≥ 14).	Once weekly for 12 weeks up to 6 months	Usual care	Severity of depression	26 weeks
**Freedland et al. 2022**	NCT02997865	139	USA	(1) Medically treated patients had a New York Heart Association class I-III HF diagnosis.	For the first eight weeks, sessions were conducted every week.	Usual care	Severity of depression	32 weeks
**Hwang et al. 2022**	NCT01937936	26	USA	(1) Patients had to be over the age of 21. (2) Patients with a primary or secondary diagnosis of HF NYHA class II-IV) (3) Patients with a family member providing care at home and be able to converse in English.	Once weekly for eight weeks	General Health Education	Salivary cortisol	26 weeks
**Khayati et al. 2020**	IRCT20160924029954N9	80	Iran	(1) Patients with heart failure who had been hospitalized. (2) Detection of heart failure based on patient data (3) BDI score of at least 21, depression diagnosis based on MSE by clinical psychologist	Five sessions	Conventional training	Depressive symptoms and severity of depression	8 weeks
**Moradi et al. 2022**	N/R	66	Iran	(1) According to the AHA, inclusion criteria included age range of 45–70 years, fluency in the Persian language, and stage B or higher heart failure.	Three times a week for 3 weeks	To comply with ethical standards, control group patients had 4 online CBT training sessions.	Death anxiety and depression	4 weeks

**CBT**: Cognitive Behavioral Therapy, **NYHA**: New York Heart Association, **HF**: Heart Failure, **AHA**: American Heart Association, **PDI**: Beck Depression Inventory, **MSE**: Mental Status Examination, **N/R**: not reported

**Table 2:** Baseline Characteristics of the included studies

Study ID	Study arms	Sample	Age, years, M ± SD	Sex, male (%)	Body mass index, Kg/m2, M ± SD	Married, n (%)	Employed, n (%)	Education (≥ 12 y), n (%)	Income (< 30,000 $/y), n (%)	NYHA classification, n (%)				Most common comorbidities, n (%)							Antidepressant medication use at admission, n (%)
										I	II	III	IV	Previous myocardial infarctions	Diabetes	Prior stroke	COPD	Hypertension	History of renal disease	History of atrial fibrillation	
**Cajanding et al. 2016**	CBT	52	Reported as age groups	33 (69%)	NR	36 (75%)	NR	15 (31%)	NR	7 (15%)	20 (42%)	19 (40%)	2 (4%)	23 (48%)	9 (17.3%)	6 (11.5%)	4 (7.7%)	19 (36.5%)	NR	NR	NR
	Control	48		34 (65%)	NR	39 (75%)	NR	16 (31%)	NR	5 (10%)	23 (44%)	21 (40%)	3 (6%)	19 (36.5%)	8 (15.4%)	4 (7.7%)	4 (7.7%)	18 (34.6%)	NR	NR	NR
**Dekker et al. 2012**	CBT	21	68 ± 10	11 (52%)	28.2 ± 6.4	14 (66.7%)	2 (10%)	5 (24%)	NR	0 (0%)	6 (29%)	15 (71%)	0 (0%)	8 (38%)	9 (43%)	4 (19%)	12 (57%)	NR	9 (43%)	8 (38%)	7 (33%)
	Control	21	64 ± 12	12 (57%)	33.8 ± 9.7	10 (48%)	1 (5%)	9 (45%)	NR	0 (0%)	2 (10%)	16 (76%)	3 (14%)	9 (43%)	10 (48%)	4 (19%)	5 (25%)	NR	7 (33%)	11 (52%)	8 (38%)
**Freedland et al. 2015**	CBT	79	56.2 ± 11.5	39 (49.4%)	34.7 ± 7.9	34 (43.0%)	17 (21.5%)	67 (84.8%)	34 (49.3%)	46 (58.2%)		33 (41.8%)	0 (0%)	25 (31.7%)	27 (34.2%)	NR	11 (13.9%)	54 (68.4%)	NR	26 (32.9%)	26 (32.9%)
	Control	79	55.5 ± 10.9	46 (58.2%)	32.6 ± 7.8	40 (50.6%)	17 (21.5%)	68 (86.1%)	32 (48.5%)	45 (57.0%)		34 (43.0%)	0 (0%)	24 (31.6%)	33 (41.8%)	NR	18 (22.8%)	59 (74.7%)	NR	30 (38.0%)	26 (32.9%)
**Freedland et al. 2022**	CBT	69	58.0 ± 11.5	36 (52.2%)	NR	26 (37.7%)	NR	17 (24.6%)	35 (50.7%)	44 (63.8%)		NR	NR	28 (40.6%)	28 (40.6%)	NR	14 (20.3%)	56 (81.2%)	21 (30.4%)	23 (33.3%)	31 (44.9%)
	Control	70	58.3 ± 12.2	35 (50%)	NR	27 (38.6%)	NR	14 (20.0%)	30 (42.9%)	47 (67.1%)		NR	NR	21 (30.0%)	35 (50.0%)	NR	21 (30.0%)	61 (87.1%)	12 (17.1%)	28 (40.0%)	31 (44.3%)
**Hwang et al. 2022**	CBT	14	66.54 ± 11.33	9 (64.3%)	NR	11 (78.6%)	3 (21.4%)	5 (35.7%)	3 (21.4%) *	0 (0.0%)	6 (42.9%)	6 (42.9%)	2 (14.3%)	NR	NR	NR	NR	NR	NR	NR	NR
	Control	12	67.41 ± 17.14	3 (25%)	NR	8 (66.7%)	1 (8.3%)	9 (75.0%)	3 (25.0%) *	0 (0.0%)	6 (50%)	6 (50%)	0 (0.0%)	NR	NR	NR	NR	NR	NR	NR	NR
**Khayati et al. 2020**	CBT	40	53.08 ± 10.11	22 (55%)	NR	36 (90%)	14 (35%)	10 (25%)	NR	NR	NR	NR	NR	NR	NR	NR	NR	NR	NR	NR	NR
	Control	40	51.63 ± 8.33	24 (60%)	NR	35 (87.5%)	13 (32.5%)	12 (30%)	NR	NR	NR	NR	NR	NR	NR	NR	NR	NR	NR	NR	NR
**Moradi et al. 2022**	CBT	33	58.70 ± 7.91	18 (54.5%)	NR	22 (66.7%)	4 (12.1%)	5 (15.2%)	NR	NR	NR	NR	NR	NR	NR	NR	NR	NR	NR	NR	NR
	Control	33	58.24 ± 8.32	20 (60.6%)	NR	25 (75.8%)	6 (18.1%)	2 (6.1%)	NR	NR	NR	NR	NR	NR	NR	NR	NR	NR	NR	NR	NR

**CBT**: Cognitive behavioral therapy, **NYHA**: New York Heart Association, **NR**: not reported, **COPD**: chronic obstructive pulmonary disease

### Quality assessment results

**Fig. 2:**
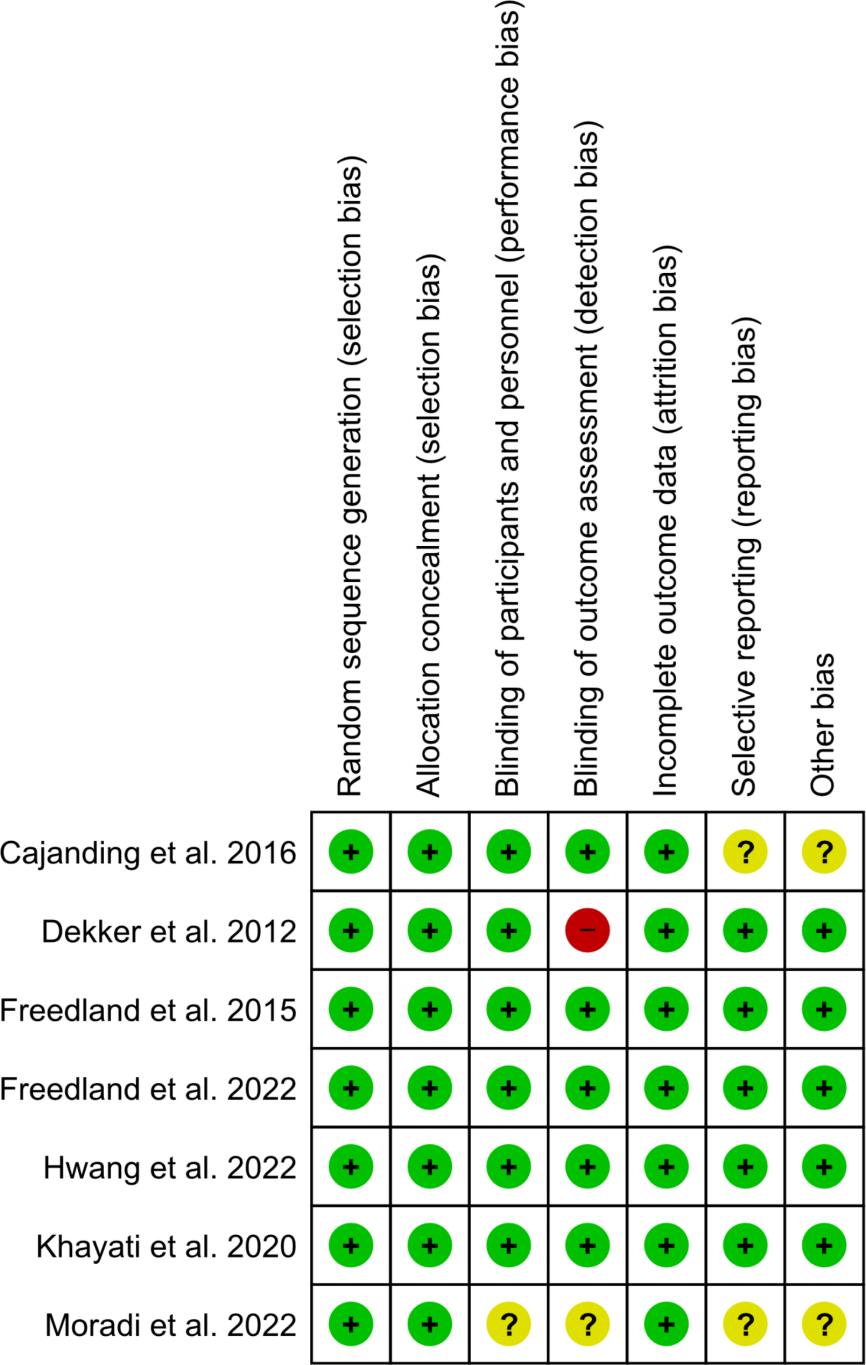
Cochrane risk of bias assessment of the include studies

Regarding selection bias, all studies showed a low risk of bias in both random sequence generation and allocation concealment. Regarding performance bias, all studies were judged as low, except Moradi et al. was unclear [24]. Regarding detection bias, all studies showed a low risk of bias, except Moradi et al. was unclear [24], and Dekker et al. was unblinded [20]. The attrition bias was judged as low in all studies. Regarding selective reporting, all studies showed a low risk of bias, except Cajanding et al. and Moradi et al. were unclear [19, 24]. Finally, no other biases were detected in all studies, except in Cajanding et al. and Moradi et al. were unclear as neither trial protocol was reported [19, 24] (Figure 2).

### Outcomes

#### Beck Depression Inventory (BDI-II)

**Fig. 3:**
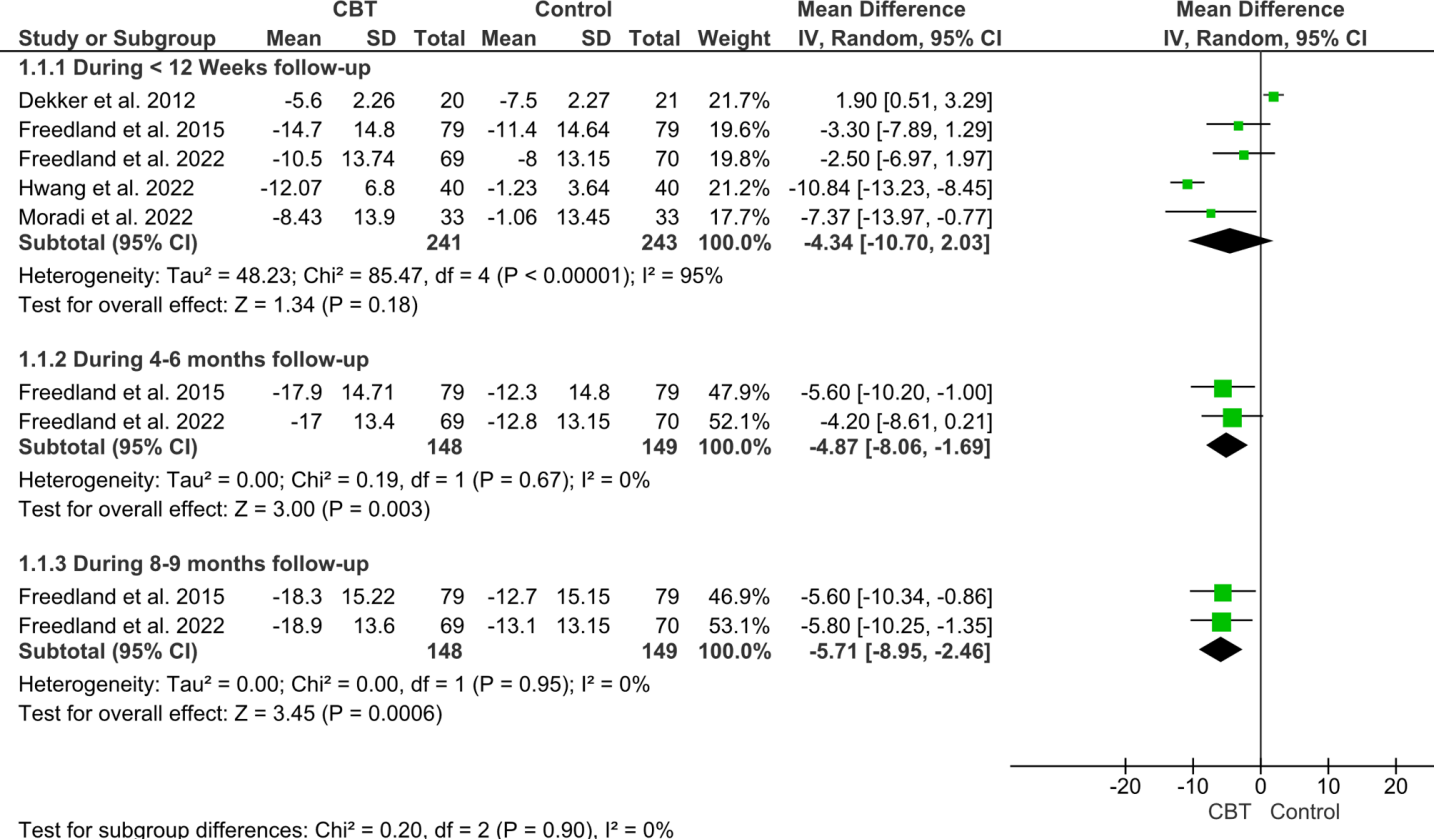
Forest plot showing the mean difference (MD) and 95% confidence interval (CI) in Beck Depression Inventory (BDI-II).

The pooled analysis of five included studies with a total sample of 484 patients showed no significant difference between CBT and the control group after the first reported follow-up (less than 12 weeks follow-up) as follows (MD = -4.34; 95% CI [-10.70, 2.03], *P = 0.18*). The pooled studies data were heterogenous (P ˂ 0.00001, I^2^ = 95%). After conducting a sensitivity analysis (leave one out meta-analysis test) to solve the heterogeneity and make sure that these results are statistically not robust, we found no statistical robustness in the outcome result; however, the significant high heterogeneity could be resolved due to high variations between the first reported follow-up between the pooled studies (Fig. 3).

However, regarding change in BDI-II after 4 to 6 months follow-up, and after 8 to 9 months follow-up, The CBT intervention group showed a significant reduction in BDI-II compared to the control group as following, (MD = -4.87, 95% CI: [-8.06; -1.69], P *= 0.003*), and (MD = -5.71, 95% CI: [-8.95; -2.46], *P = 0.0006*) respectively. The data was homogenous in both analyses as follows (P = 0.67, I^2^ = 0%) and (P = 0.95, I^2^ = 0%) respectively (Fig. 3).

#### Beck anxiety inventory (BDI-I)

The pooled effect estimates of included studies with a total sample of 297 patients showed no significant difference between CBT and the control group during the first reported follow-up duration (less than 12 weeks follow-up) (MD = -1.78, 95%CI: [-4.87; 1.31], *P = 0.26*). The pooled studies were homogenous (P = 0.64, I^2^ = 0).

**Fig. 4:**
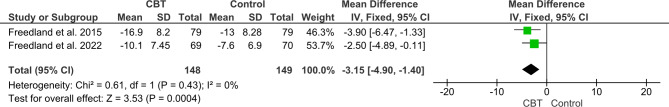
Forest plot showing the mean difference (MD) and 95% confidence interval (CI) in Beck Anxiety Inventory (BDI-I).

Regarding change in BDI-I after 4–6 months, and after 8–9 months, the CBT significantly decreased BDI-I compared to control as following (MD = -3.54, 95% CI: [-6.64; -0.45], *P = 0.02*), and (MD=-3.9; 95% CI: [-7.05; -0.74], *P = 0.02*) *respectively*. The pooled studies were homogenous in both follow-up durations as follows (P = 0.71, I^2^ = 0%) and (P = 0.73, I^2^ = 0) respectively (Fig. 4).

#### Hamilton Rating Scale for Depression (HRSD-17)

**Fig. 5:**
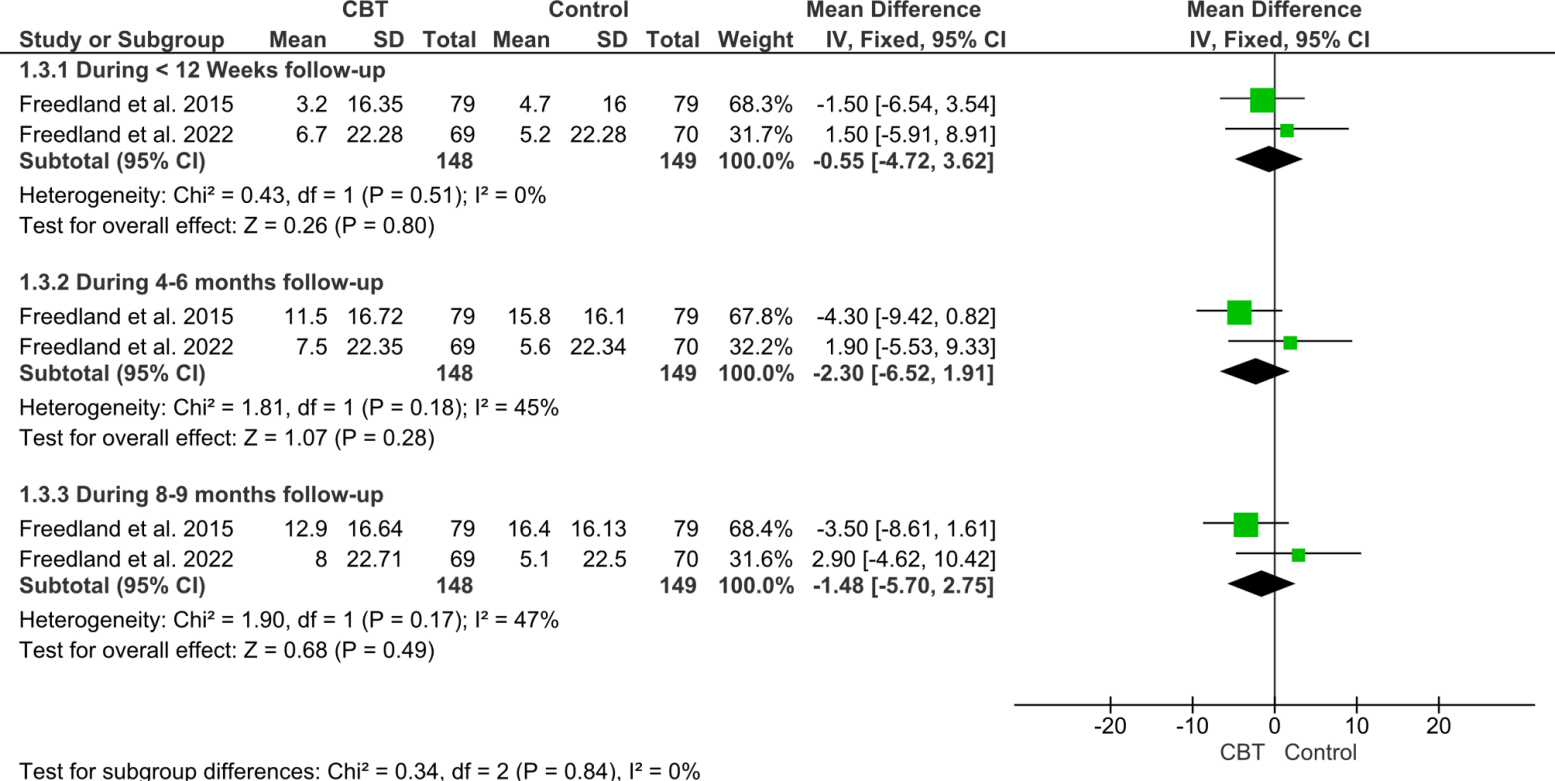
Forest plot showing the mean difference (MD) and 95% confidence interval (CI) in Hamilton Rating Scale for Depression (HRSD-17)

Regarding the HRSD-17 after 4–6 months follow-up duration, the pooled analysis of 297 patients showed a significant HRSD-17 reduction in the CBT group compared to controls (MD = -3.15, 95% CI: [-4.9; -1.4], *P = 0.0004*). The pooled data were homogenous (P = 0.43, I^2^ = 0) and no heterogeneity detected between the pooled studies (Fig. 5).

#### Self-care of heart failure index (SCHFI) maintenance

**Fig. 6:**
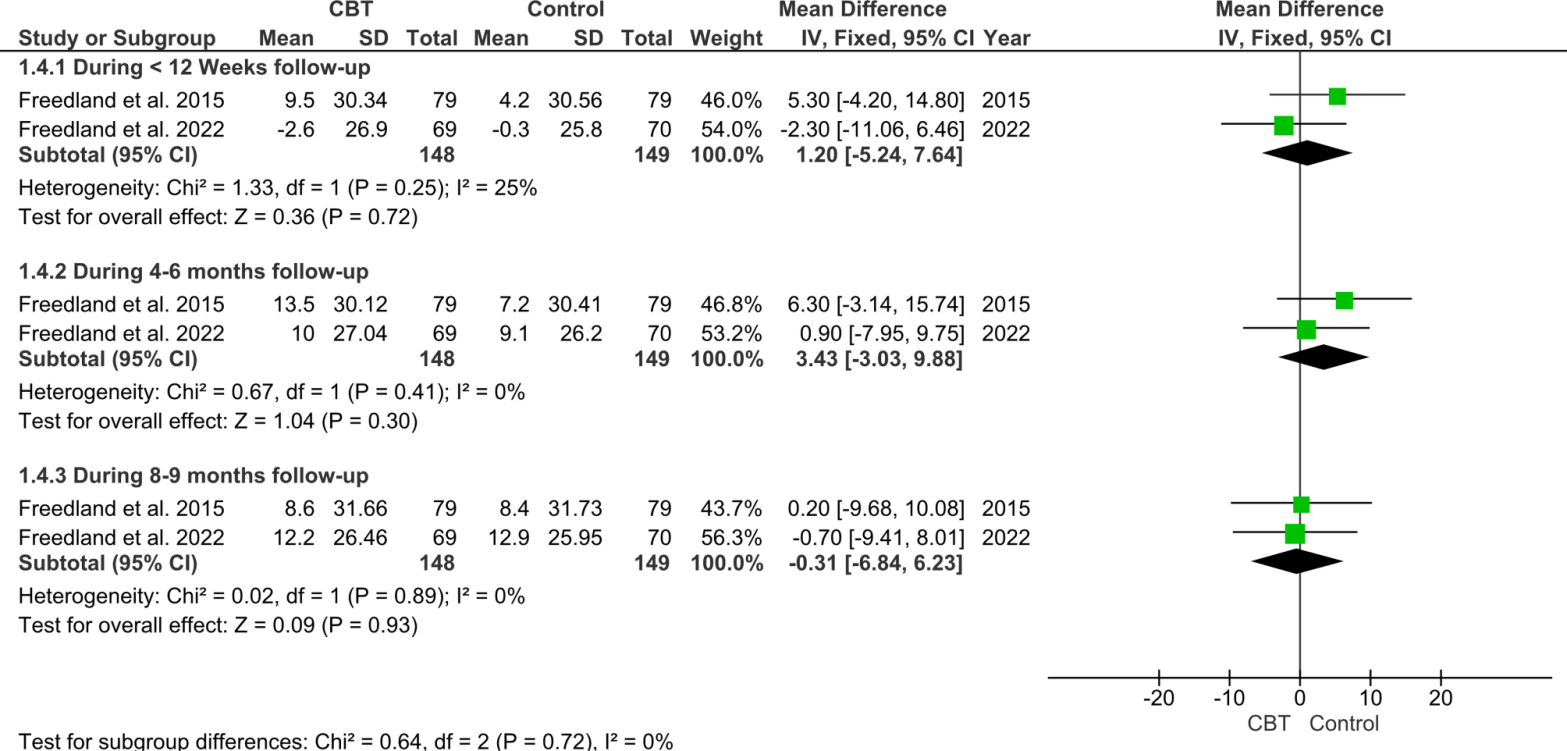
Forest plot showing the mean difference (MD) and 95% confidence interval (CI) in Self-Care of Heart Failure Index (SCHFI) Maintenance

Regarding the SCHFI maintenance component, the pooled analysis showed an insignificant difference (MD = -1.44; 95% CI: [-3.86, 0.99], P = 0.25) between CBT and Control groups with no favorable effects of CBT group either during < 12 months follow-up (MD = -0.55; 95% CI: [-4.27, 3.62], P = 0.8), 4–6 months follow-up (MD = -2.3; 95% CI: [-6.52, 1.91], P = 0.28), or 8–9 follow-up (MD = -1.48; 95% CI: [− 5.70, 2.75], P = 0.49) (Fig. 6).

#### Self-care of heart failure index (SCHFI) confidence

**Fig. 7:**
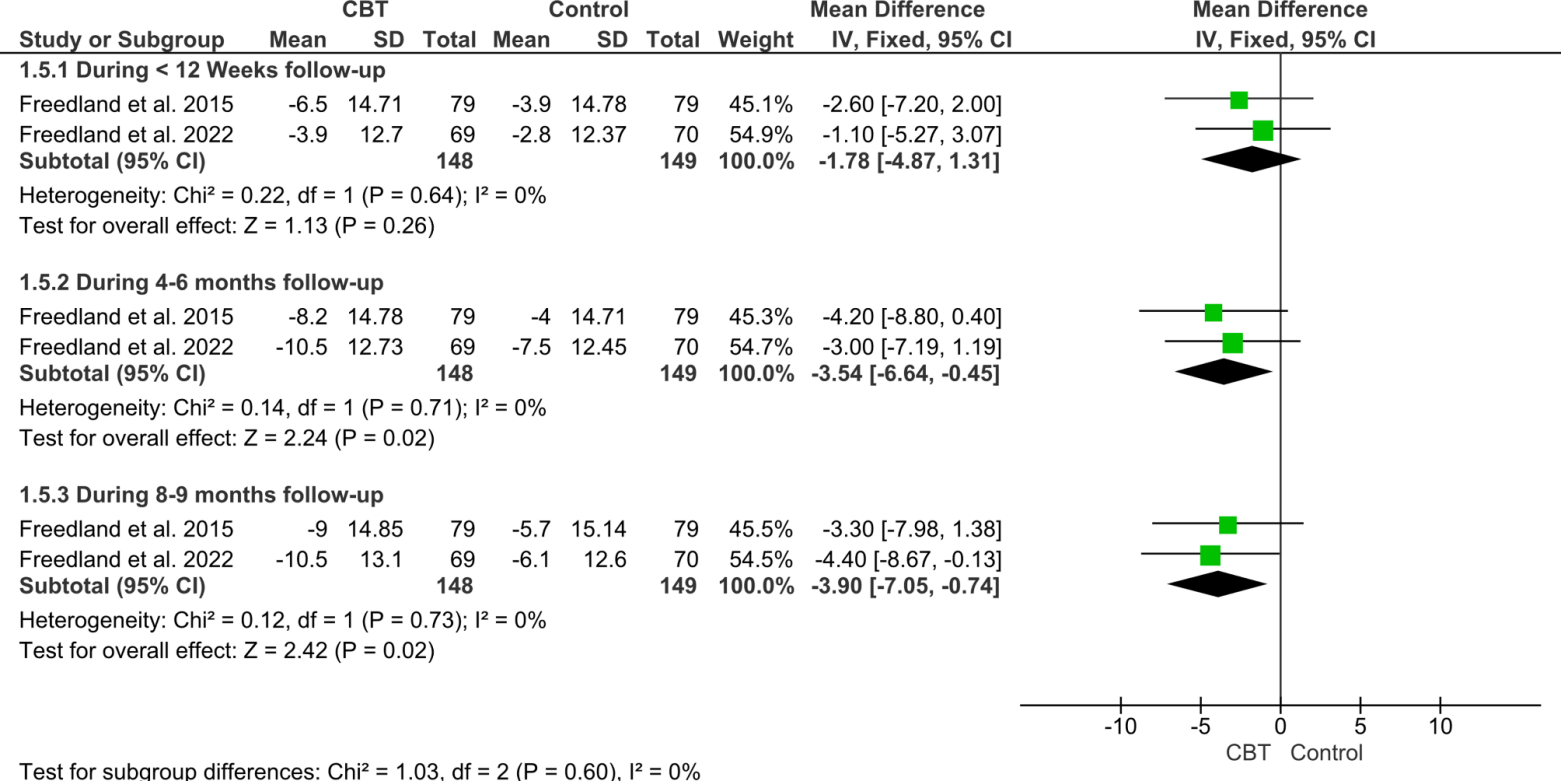
Forest plot showing the mean difference (MD) and 95% confidence interval (CI) in Self-Care of Heart Failure Index (SCHFI) Confidence

Regarding the SCHFI confidence component, the pooled analysis showed an insignificant difference (MD = 1.45; 95% CI: [-2.29, 5.19], P = 0.45) between CBT and Control groups with no favorable effects of CBT group either following < 12 months follow-up (MD = 1.20; 95% CI: [-5.24, 7.64], P = 0.72), 4–6 months follow-up (MD = 3.43; 95% CI: [-3.03, 9.88], P = 0.3), or 8–9 follow-up (MD = -0.31; 95% CI: [-6.84, 6.23], P = 0.93) (Fig. 7).

#### Kansas City Cardiomyopathy Questionnaire

The meta-analysis of the two included studies with a total sample of 297 patients showed no significant difference between CBT and the control group during the first reported follow-up duration (less than 12 weeks follow-up) (MD = 4.9; 95%CI: [-1.29; 11.09], *P = 0.12*) and after 4–6 months (MD = 4.72; 95% CI: [-1.37, 10.81], *P = 0.13*). The pooled studies were homogenous (P = 1.0, I^2^ = 0, (P = 0.35, I^2^ = 0) respectively.

**Fig. 8:**
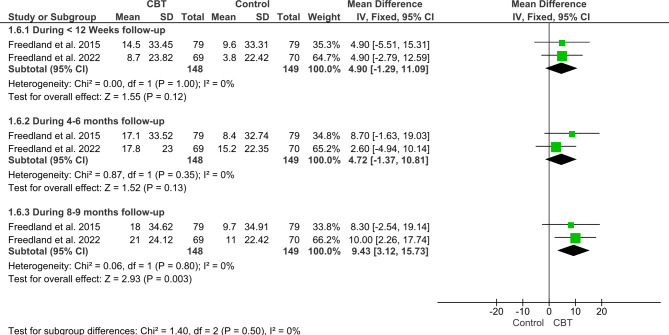
Forest plot showing the mean difference (MD) and 95% confidence interval (CI) in Kansas City Cardiomyopathy Questionnaire

On the other hand, regarding the change in KCCQ after and after 8–9 months, CBT significantly increased KCCQ compared to control as following (MD = 9.43; 95%CI: [3.12, 15.73], *P = 0.003*). *The* pooled studies were homogenous (P = 0.80, I^2^ = 0) with no heterogeneity detected (Fig. 8).

#### Systematic review and qualitative synthesis

Cajanding et al. study was not included in the meta-analysis as they reported only post-treatment results, so we could not calculate the change like other studies [19]. According to the study’s findings, cognitive-behavioral therapy administered by nurses significantly improved patients’ subjective well-being, sense of control over their lives, and overall outlook on life in Filipinos with HF. Patients with this heart ailment were encouraged to use this intervention as part of their best care.

## Discussion

Despite the strong evidence that depressive HF patients have a poorer prognosis, there is little data to support the therapeutic value of psychological interventions [26]. In an attempt to address this gap, our meta-analysis evaluated the impact of CBT on self-care, anxiety, and depression in HF patients. Our findings revealed that CBT had no significant short-term (less than 3 months) impact on HF patients’ self-care, depression, or anxiety. However, during the long term (4–9 months), CBT considerably decreased anxiety and depression versus control scenarios, greatly enhancing the quality of life. Therefore, longer follow-up and treatment durations appeared to be a key element in treatment effectiveness.

Investigations have shown that CBT has a strong therapeutic impact on psychological outcomes. This was supported by an earlier comprehensive analysis evaluating psychological interventions’ implications for coronary heart disease patients [27]. Additionally, it is fascinating to consider how culture may influence the intervention strategy. While Chinese people often see mental illnesses as a stigma and show more somatic symptoms when they are in a psychological problem, psychotherapy with a somatic symptom-management component appears to be more accepting and efficient in enhancing the psychological health of this population [28–30]. In contrast, Western peers developed more empowerment-based and cognitive-oriented strategy to lessen psychological suffering [20, 21].

A recent pooled analysis [14] found that CBT was more successful than conventional treatment in treating depressive patients with HF. They also found that although they were highly chosen patients at chosen facilities with various comparators and subjective outcome measures, this result persisted 3 months after the CBT sessions were finished [14]. At 3 months, two RCTs showed a higher improvement in depression ratings [20, 21]. This could be because of the duration and frequency of CBT sessions in these RCTs, as the sessions conducted on a weekly basis over time were more effective [21, 31] than a single session [20]. Based on the primary (BDI-II) measure of depression, our pooled analysis found that longer follow-up and treatment durations appeared to be a key element in treatment effectiveness. We found that CBT had no significant short-term (less than 3 months) effects on depression, but in the long-term (4–9 months), CBT significantly reduced depression.

Besides the positive effect of CBT on depression, we found that it significantly reduces HF patients’ anxiety symptoms. Through CBT involvement, we could assist the patients in avoiding exaggerated and catastrophic thinking about the illness and guide them to positive thoughts because battling negative beliefs encourages patients to grow a strong spirit and become more motivated to recover and deal with the illness and its effects [24]. A prior RCT reported that the death anxiety was substantially reduced in the CBT arm as opposed to the control arm [24]. Another study verified that CBT reduces anxiety, improves the quality of life associated with HF, and is helpful for treating severe depression in HF patients [21].

Self-care for HF patients involves effort, participation, and the capacity to plan everyday activities. Fatigue, despair, and poor attention are the depressive symptoms that interfere with these capacities and make it challenging to practice adequate self-care. It is thus anticipated that effective therapy for depression will increase patients’ capacity to gain from a customized HF self-care intervention. Self-care education based on CBT concepts encourages HF patients with depression to stick with self-care practices [22].

According to research by Zakerimoghadam et al., [32] education aimed at changing people’s misconceptions about the illness has a good and noticeable effect on the self-care of HF patients [32]. Similarly, Graven and Grant reported that self-confidence, which is significantly diminished in depression, does not only function as an independent but also a mediator factor for self-care in HF patients and that its reinforcement promotes self-care commitment behaviors while helping to treat depression [33]. Our pooled analysis was in line with these findings; however, the results were still insignificant concerning the self-care outcomes.

The impacts of CBT on a number of secondary outcomes, such as anxiety, exhaustion, HF-related quality of life, and contentment with social activities and roles, show that CBT may potentially have other advantages for HF patients [34]. According to a recent meta-analysis, CBT initially improved quality of life more than conventional care (3 months CBT phase), but there was no indication of a difference between the CBT and control groups at later periods [14]. In light of our pooled analysis, we found a considerable increase in HF-related quality of life in the long term (8–9 months), which was consistent with the preintervention/postintervention change in the CBT group on the KCCQ.

Previous guidelines indicated that the data used to assess the benefits of CBT on depression do not just apply to patients with HF; they also apply to people with depression and people with long-term physical health issues. This could be because there was not enough evidence on adopting CBT for depression in HF when these recommendations were published [35]. A definite recommendation that CBT should be taken into consideration in depressed HF patients is made in the 2016 Scottish Intercollegiate Guidelines Network (SIGN) for treating chronic HF [36]. The basis for this recommendation’s support is a single RCT and a comprehensive analysis of several interventions that only included one trial on CBT [37]. Thus, the current pooled analysis builds on earlier research and offers a thorough examination of the presently available data regarding the impact of CBT on depressed HF patients.

### Limitations

Several limitations must be addressed, notwithstanding the meta-analysis results. The scanning turned up just seven RCTs, highlighting the lack of experimental trials evaluating the effectiveness of CBT in people with HF. Due to the nature of CBT therapies, performance bias was inevitable in the RCTs since participants were not blinded. Additionally, methodological research quality was questionable due to a lack of data. We also cannot ignore that self-reported surveys may have added social desirability bias when evaluating the results. The moderate to high heterogeneity among the selected trials was another drawback of this analysis, which may have affected the accuracy and repeatability of the findings. Lastly, another possible issue was the difficulty in ascertaining whether any placebo effects instead of the specific therapies contributed to a favorable outcome. As a result, it is essential to use caution when interpreting review findings.

## Conclusion

CBT had no significant short-term (less than 3 months) impact on HF patients’ self-care, depression, or anxiety. However, during the long term (4–9 months), CBT considerably decreased anxiety and depression versus control scenarios, greatly enhancing the quality of life. Therefore, longer follow-up and treatment durations appeared to be a key element in treatment effectiveness. To confirm the long-term effects and determine the cost-effectiveness of a CBT approach in depressed HF patients, more extensive and reliable RCTs are required.

Finally, due to the increased frequency of psychiatric conditions in people with HF, it is advised that healthcare professionals screen these patients. The use of CBT concepts in treatment plans is recommended to create a synergistic intervention that might significantly improve HF patients’ physical and mental health.

## Data Availability

All data generated or analyzed during this study are included in this published article. Raw data used for analysis are available upon reasonable request from the corresponding author.
